# A protocol of systematic review and meta-analysis of Mozart's Music for drug-resistant epilepsy

**DOI:** 10.1097/MD.0000000000021090

**Published:** 2020-07-17

**Authors:** Ke-Jian Wang, Shi-Hua Zhang, Jia-Nan Yu, Guang-Tao Sun, Shu-Xin Dong

**Affiliations:** a Fourth Ward of Neurology Department; b Department of Neurosurgery; c Third Ward of Neurology Department; d Rehabilitation Ward of Neurology Department, First Affiliated Hospital of Jiamusi University, Jiamusi, China.

**Keywords:** drug-resistant epilepsy, Mozart music, effectiveness

## Abstract

**Background:**

This study will aim to assess the effectiveness of Mozart's Music (MM) for the management of patients with drug-resistant epilepsy (DRE).

**Methods:**

In this study, we will search MEDLINE, EMBASE, Cochrane Library, Web of Science, CINAHL, Chinese Scientific Journal Database Information, WANGFANG, and China National Knowledge Infrastructure from their inauguration to March 1, 2020 without language and publication time restrictions. We will also identify other literature resources, such as reference lists of any related reviews. Trial quality will be examined by Cochrane risk of bias tool; reporting bias will be identified by a funnel plot and Egger test; and statistical analysis will be undertaken by RevMan 5.3 software.

**Results:**

This study will summarize high quality randomized controlled trials to appraise the effectiveness and safety of MM for the treatment of patients with DRE.

**Conclusions:**

The findings of this study will supply evidence to judge whether MM is effective on DRE at evidence-based medicine level.

**Study registration number:**

CRD42020170512.

## 1 Introduction

Epilepsy is a chronic neurological disorder, affecting more than 70 million people worldwide, with an estimated prevalence of 0.5% to 1%.^[1–5]^ Of those, patients with epilepsy who fail to respond to antiepileptic drug treatment are defined as having drug-resistant epilepsy (DRE).^[6–9]^ It has been reported that about 30% patients with epilepsy suffer from DRE.^[10]^ It is often associated with morbidity, functional impairment, and poor quality of life.^[11–13]^ Thus, it is very important to find alternative therapy to manage such condition.^[14,15]^

Mozart Music (MM) is reported to treat patients with DRE.^[16–22]^ However, no systematic review is conducted to assess the effectiveness of MM in treating DRE. Therefore, this study aims to systematically and comprehensively evaluate the effectiveness and safety of MM for the treatment of patients with DRE.

## 2 Methods

### 2.1 Study registration

This study was registered on PROSPERO (CRD42020170512). It is organized following the guidelines of the preferred reporting items for systematic reviews and meta-analysis protocol statement.^[23,24]^

### 2.2 Ethics and dissemination

It is not necessary to provide ethical approval, because this study will not collect original patient data. We will publish this study in a scientific peer-reviewed journal or a conference meeting.

### 2.3 Criteria for including studies

#### 2.3.1 Types of studies

This study will only consider randomized controlled trials (RCTs) assessing the effectiveness of MM for the treatment of patients with DRE. We will not consider any other studies, except RCTs.

#### 2.3.2 Types of interventions

All patients in the experimental group received MM alone as their treatment.

All participants in the control group underwent any treatments, such as placebo, sham intervention, medication, and alternative therapy. However, we will exclude any combinations with MM as a control intervention.

#### 2.3.3 Types of patients

Any participants who were diagnosed as DRE regardless their race, gender, age, severity and duration of DRE will be included.

#### 2.3.4 Types of outcome measurements

Outcomes include seizure freedom, frequency of seizures, quality of life, number of emergency visits within 1 week or month, and any adverse events.

### 2.4 Data resources and searches

We will carry out a comprehensive search of literatures that have been published from their inauguration to March 1, 2020 without language and publication time limitations in MEDLINE, EMBASE, Cochrane Library, Web of Science, CINAHL, Chinese Scientific Journal Database Information, WANGFANG, and China National Knowledge Infrastructure. The preliminary search strategy for MEDLINE is presented (Table 1). We will adapt identical search strategies for other electronic databases.

**Table 1:**
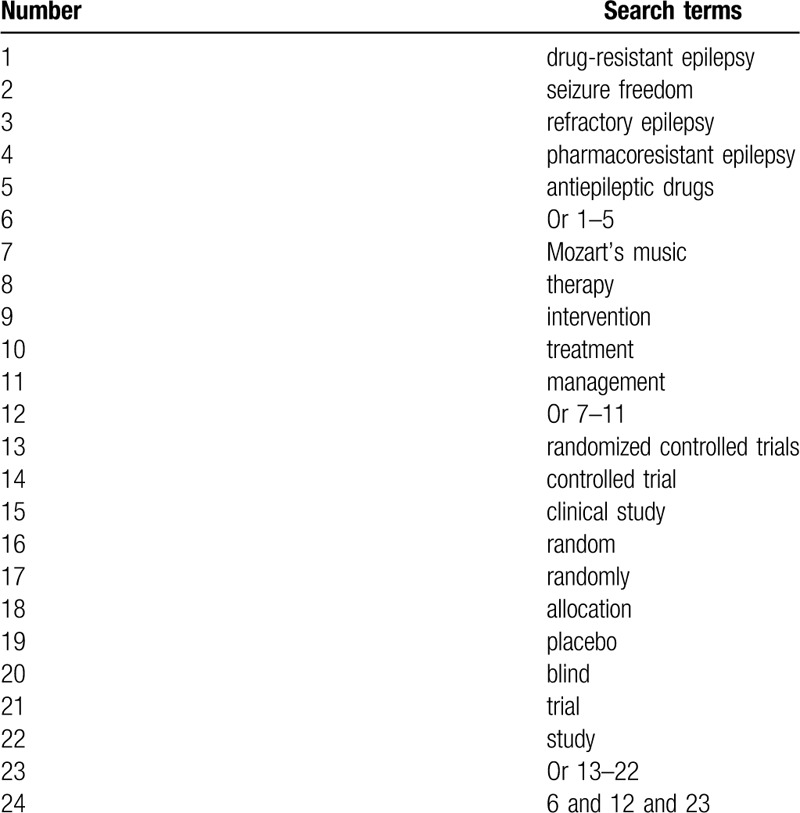
Detailed search strategy of MEDLINE.

Besides the above electronic databases, we will search other literature resources, such as conference proceedings, dissertations, and reference lists of any related reviews.

### 2.5 Data collection and analysis

#### 2.5.1 Study selection

All searched records will be imported into Endnote X7 to eliminate any duplication retrieved from the literature searches. Two authors will independently check the titles and abstracts of literatures to determine eligibility for inclusion in this study. If the information needed to judge eligibility with insufficient details, we will obtain full-texts to further identify if they meet all inclusion criteria. All excluded studies will be noted with specific reasons. Any differences will be resolved by a third author through discussion, and a final decision will be reached. The process of study selection will be presented in a flow diagram.

#### 2.5.2 Data collection

Two authors will independently extract the following information utilizing an advance-designed data collection form. Any inconsistencies will be figured out by consulting another experienced author. The collected information includes publication information (such as title, author, time of publication, et al), patient characteristics (such as age, race, gender, diagnostic criteria, et al), trial setting, trial design (such as sample size, randomization details, et al), interventions, controls, outcome measurements, safety, results, findings, and any other necessary information.

#### 2.5.3 Missing data dealing with

Any insufficient or missing data will be request by contacting primary authors through email. If we cannot receive those data, we will employ an intention-to-treat analysis based on the available data.

#### 2.5.4 Risk of bias assessment

Two authors will independently investigate the risk of bias of each eligible trial using internationally recognized Cochrane risk of bias tool, which has 7 sectors, and each one is graded as low, unclear or high risk of bias. Any disagreements will be resolved by discussion with another experienced author when necessary.

#### 2.5.5 Subgroup analysis

A subgroup analysis will be carried out to examine any sources of obvious heterogeneity in accordance with the types of interventions, controls, and outcome measurements.

#### 2.5.6 Sensitivity analysis

A sensitivity analysis will be performed to check the stability of study findings by removing low quality trials.

#### 2.5.7 Reporting bias

We will inspect reporting bias by a funnel plot and Egger regression test if over 10 trials are included.^[25,26]^

#### 2.5.8 Quality of evidence

Quality of evidence of each outcome will be scrutinized by 2 independent authors utilizing grading of recommendations assessment development and evaluation.^[27]^ Any different opinions will be settled down by another author through discussion.

### 2.6 Data synthesis

This study will employ RevMan 5.3 software to perform all statistical analysis. We will present all continuous variables as mean difference or standardized mean difference and 95% confidence intervals (CIs), and all dichotomous variables as risk ratio and 95% CIs. Statistical heterogeneity will be identified using *I*^*2*^ statistics. Low heterogeneity is defined as *I*^*2*^ ≤50% and a fixed-effects model will be applied, while high heterogeneity is defined as *I*^*2*^ > 50%, and a random-effects model will be employed. If sufficient trials are included, meta-analysis will be conducted under the likely circumstance in the absence of significant heterogeneity. If there is high heterogeneity, we will carry out subgroup analysis to explore the sources of significant heterogeneity.

## 3 Discussion

Studies suggested the effectiveness and safety of MM in treating patients with DRE.^[16–22]^ So far, no systematic review has been performed to evaluate the comparative effectiveness and safety of MM for DRE. Therefore, it is very essential to judge its comparative effectiveness in treating DRE.

To our best knowledge, this is the first study to assess the effectiveness and safety of MM in treating patients with DRE. On the basis of comparative effectiveness evidence, this study will target to summarize up-to-date evidence of MM in treating DRE. Its findings may help patients, clinicians, and future researches.

## Author contributions

**Conceptualization:** Ke-jian Wang, Jia-nan Yu.

**Data curation:** Shi-hua Zhang, Shu-xin Dong.

**Formal analysis:** Ke-jian Wang, Jia-nan Yu, Shu-xin Dong.

**Investigation:** Shu-xin Dong.

**Methodology:** Ke-jian Wang, Shi-hua Zhang, Jia-nan Yu, Guang-tao Sun.

**Project administration:** Shu-xin Dong.

**Resources:** Ke-jian Wang, Shi-hua Zhang, Jia-nan Yu, Guang-tao Sun.

**Software:** Ke-jian Wang, Shi-hua Zhang, Guang-tao Sun.

**Supervision:** Shu-xin Dong.

**Validation:** Ke-jian Wang, Shi-hua Zhang, Jia-nan Yu, Guang-tao Sun, Shu-xin Dong.

**Visualization:** Ke-jian Wang, Guang-tao Sun, Shu-xin Dong.

**Writing – original draft:** Ke-jian Wang, Shi-hua Zhang, Jia-nan Yu, Guang-tao Sun, Shu-xin Dong.

**Writing – review & editing:** Ke-jian Wang, Shi-hua Zhang, Shu-xin Dong.
